# Convergence theorems for split feasibility problems on a finite sum of monotone operators and a family of nonexpansive mappings

**DOI:** 10.1186/s13660-018-1799-3

**Published:** 2018-08-08

**Authors:** Narin Petrot, Montira Suwannaprapa, Vahid Dadashi

**Affiliations:** 1 Department of Mathematics, Faculty of Science, Naresuan University, Phitsanulok, Thailand; 2 Department of Mathematics, Sari Branch, Islamic Azad University, Sari, Iran

**Keywords:** 26A18, 47H04, 54A20, Maximal monotone operator, Inverse strongly monotone operator, Resolvent operator, Convex feasibility problems

## Abstract

In this paper, we present two iterative algorithms for approximating a solution of the split feasibility problem on zeros of a sum of monotone operators and fixed points of a finite family of nonexpansive mappings. Weak and strong convergence theorems are proved in the framework of Hilbert spaces under some mild conditions. We apply the obtained main result for the problem of finding a common zero of the sum of inverse strongly monotone operators and maximal monotone operators, for finding a common zero of a finite family of maximal monotone operators, for finding a solution of multiple sets split common null point problem, and for finding a solution of multiple sets split convex feasibility problem. Some applications of the main results are also provided.

## Introduction

A very common problem in different areas of mathematics and physical sciences consists of finding a point in the intersection of convex sets and is formulated as finding a point $z\in H$ satisfying the property

$$\begin{aligned} z\in \bigcap_{i=1}^{M}C_{i}, \end{aligned}$$

where $C_{i}$, $i=1, \ldots, M$, are nonempty, closed, and convex subsets of a Hilbert space *H*. This problem is called the convex feasibility problem (CFP). There are various applications of CFP in many applied disciplines as diverse as applied mathematics, approximation theory, image recovery and signal processing, control theory, biomedical engineering, communications, and geophysics (see [1–7] and the references therein).

The problem of finding $z\in H_{1}$ such that $z\in C$ and $Lz\in D$ is called the split feasibility problem (SFP), where *C* and *D* are nonempty, closed, and convex subsets of real Hilbert spaces $H_{1}$ and $H_{2}$, respectively, and $L:H_{1}\rightarrow H_{2}$ is a bounded linear operator. Let $L^{-1}(D)=\{x: Lx\in D\}$, then the SFP can be viewed as a special case of the CFP since it can be rewritten as $z\in C\cap L^{-1}D$. However, the methodologies for studying the SFP are actually different from those for the CFP; see [8–14].

The theory of monotone operators has appeared as a powerful and effective tool for studying a wide class of problems arising in different branches of social, engineering, and pure sciences in a unified and general framework. There is a notion about monotone operators and it is one of generalized sums of two monotone operators; see [15, 16] and the references therein. In recent years, monotone operators have received a lot of attention for treating zero points of monotone operators and fixed point of mappings which are Lipschitz continuous; see [17–22] and the references therein. The first algorithm for approximating the zero points of the maximal monotone operator was introduced by Martinet [23]. He considered the proximal point algorithm for finding zero points of a maximal monotone operator. Then, Passty [24] introduced a forward-backward algorithm method for finding zero points of the sum of two operators. There are various applications of the problem of finding zero points of the sum of two operators; see [25–29] for example and the references therein.

Therefore, there are some generalizations of the CFP, which can be formulated in various ways such as: finding a common fixed point of nonexpansive operators, finding a common minimum of convex functionals, finding a common zero of maximal monotone operators, solving a system of variational inequalities, and solving a system of convex inequalities. Surveys of methods for solving such problems can be found in [2, 4].

Recently, some authors introduced and studied algorithms to get a common solution to inclusion problems and fixed point problems in the framework of Hilbert spaces; see [30–32]. Cho et al. [30] considered the problem of finding a common solution to the zero point problems involving two monotone operators and fixed point problems involving asymptotically strictly pseudocontractive mappings based on a one-step iterative method and proved the weak convergence theorems in the framework of Hilbert spaces.

In this paper, motivated and inspired by the above literature, we consider an iterative algorithm for finding a solution of split feasibility problem for a point in zeros of a finite sum of *α*-inverse strongly monotone operators and maximal monotone operators and fixed points of nonexpansive mappings. That is, we are going to consider the following problem: Let $H_{1}$ and $H_{2}$ be real Hilbert spaces. Let $A_{i}:H_{1}\rightarrow H_{1}$, $i=1, \ldots, M$, be $\alpha_{i}$-inverse strongly monotone operators and $B_{i}:H_{1} \rightarrow 2^{H_{1}}$, $i=1, \ldots, M$, be maximal monotone operators, $T_{j}:H_{2}\rightarrow H_{2}$, $j=1, \ldots, N$, be nonexpansive mappings, $L:H_{1}\rightarrow H_{2}$ be a bounded linear operator. We are interested in considering the problem of finding a solution $p\in H_{1}$ such that

1.1 $$\begin{aligned} p\in \Biggl( \bigcap_{i=1}^{M}(A_{i}+B_{i})^{-1}(0) \Biggr) \cap L^{-1} \Biggl( \bigcap_{j=1}^{N}F(T_{j}) \Biggr) =: \mathcal{F}, \end{aligned}$$

where $\mathcal{F}\neq \emptyset $. Weak and strong convergence theorems will be provided under some mild conditions.

The paper is organized as follows. Section 2 gathers some definitions and lemmas of geometry of Hilbert spaces and monotone operators, which will be needed in the remaining sections. In Sect. 3, we prepare an iterative algorithm and prove the weak and strong convergence theorems. Finally, in Sect. 4, the results of Sect. 3 are applied to solve CFP, multiple-set null point problems, variational inequality problems, fixed point problems, and equilibrium problems.

## Preliminaries

Throughout this paper, *H* will be a Hilbert space with norm $\|\cdot \|$ and inner product $\langle \cdot,\cdot \rangle $, respectively. We now provide some basic concepts, definitions, and lemmas which will be used in the sequel. We write $x_{n} \rightarrow x$ to indicate that the sequence $\{x_{n}\}$ converges strongly to *x* and $x_{n} \rightharpoonup x$ to indicate that $\{x_{n}\}$ converges weakly to *x*.

Let $T:H\rightarrow H$ be a mapping. We say that *T* is a Lipschitz mapping if there exists $L\geq 0$ such that

$$ \Vert Tx-Ty \Vert \leq L \Vert x-y \Vert , \quad \forall x,y\in H. $$

The number *L*, associated with *T*, is called a Lipschitz constant. If $L=1$, we say that *T* is a nonexpansive mapping, that is,

$$ \Vert Tx-Ty \Vert \leq \Vert x-y \Vert , \quad \forall x,y\in H. $$

We will say that *T* is firmly nonexpansive if

$$ \langle Tx-Ty,x-y\rangle \geq \Vert Tx-Ty \Vert ^{2}, \quad \forall x,y\in H. $$

The set of fixed points of *T* will be denoted by $F(T)$, that is, $F(T)=\{x\in H : Tx=x\}$. It is well known that if *T* is nonexpansive, then $F(T)$ is closed and convex. Moreover, every nonexpansive operator $T:H\rightarrow H$ satisfies the following inequality:

$$\begin{aligned} \bigl\langle (x-Tx)-(y-Ty),Ty-Tx\bigr\rangle \leq \frac{1}{2} \bigl\Vert (Tx-x)-(Ty-y) \bigr\Vert ^{2}, \quad \forall x,y\in H. \end{aligned}$$

Therefore, for all $x\in H$ and $y\in F(T)$,

2.1 $$\begin{aligned} \langle x-Tx,y-Tx\rangle \leq \frac{1}{2} \Vert Tx-x \Vert ^{2}, \quad \forall x,y\in H, \end{aligned}$$

see [33, 34].

### Lemma 2.1

([35])

*Let* *H* *be a real Hilbert space and* $T:H\rightarrow H$ *be a nonexpansive mapping with* $F(T)\neq \emptyset $. *Then the mapping* $I -T$ *is demiclosed at zero*, *that is*, *if* $\{x_{n}\}$ *is a sequence in* *H* *such that* $x_{n}\rightharpoonup x$ *and* $\|x_{n} -Tx_{n}\|\rightarrow 0$, *then* $x \in F(T)$.

A mapping $T :H \rightarrow H$ is called *α*-averaged if there exists $\alpha \in (0, 1)$ such that $T=(1-\alpha)I+\alpha S$, where S is a nonexpansive mapping of *H* into *H*. It should be observed that firmly nonexpansive mappings are $\frac{1}{2}$-averaged mappings.

We now recall the concepts and facts on the class of monotone operators, for both single and multi-valued operators.

An operator $A:H\rightarrow H$ is called *α*-inverse strongly monotone (*α*-ism) for a positive number *α* if

$$\begin{aligned} \langle Ax-Ay,x-y\rangle \geq \alpha \Vert Ax-Ay \Vert ^{2}, \quad \forall x,y\in H. \end{aligned}$$

### Lemma 2.2

([21])

*Let* *C* *be a nonempty*, *closed*, *and convex subset of a real Hilbert space H*. *Let the mapping* $A:C \rightarrow H$ *be* *α*-*inverse strongly monotone and* $r >0$ *be a constant*. *Then we have*

$$\begin{aligned} \bigl\Vert (I-r A)x-(I-r A)y \bigr\Vert ^{2}\leq \Vert x-y \Vert ^{2}+r(r-2\alpha) \Vert Ax-Ay \Vert ^{2} \end{aligned}$$

*for all* $x,y\in C$. *In particular*, *if* $0< r\leq 2\alpha $, *then* $I-rA$ *is nonexpansive*.

We have the following properties from [36, 37].

### Lemma 2.3

*We have*

- *The composite of finitely many averaged mappings is averaged*. *In particular*, *if* $T_{i}$ *is* $\alpha_{i}$-*averaged*, *where* $\alpha_{i} \in (0,1)$ *for* $i=1,2$, *then the composite* $T_{1}T_{2}$ *is* *α*-*averaged*, *where* $\alpha =\alpha_{1}+\alpha_{2}-\alpha_{1} \alpha_{2}$.
- *If A is* *β*-*ism and* $r\in (0,\beta ]$, *then* $T := I-rA$ *is firmly nonexpansive*.

A multifunction $B:H\rightarrow 2^{ H}$ is called a *monotone operator* if, for every $x,y\in H$,

$$\begin{aligned} \bigl\langle x^{*}-y^{*},x-y\bigr\rangle \geq 0, \quad \forall x^{*}\in B(x), \forall y^{*}\in B(y). \end{aligned}$$

A monotone operator $B:H\rightarrow 2^{ H}$ is said to be *maximal monotone*, when its graph is not properly included in the graph of any other monotone operators on the same space. For a maximal monotone operator *B* on *H*, and $\lambda >0$, we define the single-valued resolvent $J_{\lambda }^{B} : H \rightarrow D(B)$ by $J_{\lambda }^{B}=(I + \lambda B)^{-1}$. It is well known that $J_{\lambda }^{B}$ is firmly nonexpansive, and $F(J_{\lambda }^{B})=B ^{-1}(0)$.

Next, we collect some useful facts on monotone operators that will be used in our proof.

### Lemma 2.4

([38])

*Let* *C* *be a nonempty*, *closed*, *and convex subset of a real Hilbert space* *H* *and* $A:C \rightarrow H$ *be an operator*. *If* $B:H\rightarrow 2^{ H}$ *is a maximal monotone operator*, *then* $F(J_{\lambda }^{B}(I-\lambda A ))=(A+B)^{-1}(0)$.

### Lemma 2.5

([39])

*Let* $B:H\rightarrow 2^{ H}$ *be a maximal monotone operator*. *For* $\lambda >0$, $\mu >0$, *and* $x\in H$,

$$\begin{aligned} J_{\lambda }^{B} x=J_{\mu }^{B} \biggl( \frac{\mu }{\lambda } x+ \biggl( 1-\frac{ \mu }{\lambda } \biggr) J_{\lambda }^{B} x \biggr). \end{aligned}$$

For each sequence $\{x_{n}\}\subset H$, we put

$$ \omega_{w}(x_{n}):=\bigl\{ x^{*}\in H: { \text{there is a subsequence }} \{x_{n_{j}}\}\subset \{x_{n}\} { \text{ such that }} x_{n_{j}}\rightharpoonup x^{\ast } \bigr\} . $$

The following lemma plays an important role in concluding our results.

### Lemma 2.6

([37])

*Let* *C* *be a nonempty closed convex subset of a real Hilbert space* *H*. *Let* $\{x_{n}\}$ *be a sequence in* *H* *satisfying the properties*:

(i) $\lim_{n\rightarrow \infty }\|x_{n}-u\|$ *exists for each* $u\in C$;
(ii) $\omega_{w}(x_{n})\subset C$.

*Then* $\{x_{n}\}$ *converges weakly to a point in* *C*.

## Parallel algorithm

Let $H_{1}$ and $H_{2}$ be real Hilbert spaces. Let $A_{i}:H_{1} \rightarrow H_{1}$, $i=1, \ldots, M$, be $\alpha_{i}$-inverse strongly monotone operators and $B_{i}:H_{1}\rightarrow 2^{H_{1}}$, $i=1, \ldots, M$, be maximal monotone operators, $T_{j}:H_{2}\rightarrow H _{2}$, $j=1, \ldots, N$, be nonexpansive mappings, $L:H_{1}\rightarrow H_{2}$ be a bounded linear operator. We will denote by $L^{\ast }$ the adjoint operator of *L*. Let $\{\beta_{n}\}$ and $\{\lambda_{n}\}$ be sequences of positive real numbers. For $x_{1}\in H_{1}$, we introduce the following parallel algorithm:

3.1 $$\begin{aligned} \textstyle\begin{cases} y_{j,n}=x_{n}+\lambda_{n} L^{*}( T_{j}-I)Lx_{n}, \quad j=1, \ldots, N, \\ \mbox{choose} \quad j_{n}: \Vert y_{j_{n},n}-x_{n} \Vert =\max_{j=1, \ldots, N} \Vert y_{j,n}-x _{n} \Vert , \\ y_{n}=y_{j_{n},n}, \\ z_{i,n}=J_{\beta_{n}}^{B_{i}}(I-\beta_{n} A_{i})y_{n}, \quad i=1, \ldots, M, \\ \mbox{choose} \quad i_{n}: \Vert z_{i_{n},n}-x_{n} \Vert =\max_{i=1, \ldots, M} \Vert z_{i,n}-x _{n} \Vert , \\ x_{n+1}=z_{i_{n},n}. \end{cases}\displaystyle \end{aligned}$$

We start by some lemmas.

### Lemma 3.1

*Let* $\alpha =\min \{\alpha_{1}, \ldots,\alpha_{M}\}$. *If*

(i) $\{\beta_{n}\}\subset (0, 2\alpha)$ *and*
(ii) $\{\lambda_{n}\}\subset ( a, \frac{1}{\|L\|^{2}} ) $ *for some* $a>0$,

*then the sequences* $\{x_{n}\}$ *and* $\{y_{n}\}$ *generated by* (3.1) *are bounded*.

### Proof

Let $u\in \mathcal{F}$. We have

3.2 $$\begin{aligned} \Vert y_{n}-u \Vert ^{2} =& \bigl\Vert x_{n}+\lambda_{n} L^{*}( T_{j_{n}}-I)Lx_{n}-u \bigr\Vert ^{2} \\ =& \Vert x_{n}-u \Vert ^{2}+2\lambda_{n} \bigl\langle x_{n}-u, L^{*}( T_{j_{n}}-I)Lx _{n}\bigr\rangle \\ &{}+\lambda_{n}^{2} \bigl\Vert L^{*}( T_{j_{n}}-I)Lx_{n} \bigr\Vert ^{2}. \end{aligned}$$

By (2.1), we get

3.3 $$\begin{aligned}& \bigl\langle x_{n}-u,L^{*}(T_{j_{n}}-I)Lx_{n} \bigr\rangle \\& \quad =\langle Lx_{n}-T _{j_{n}}Lx_{n}+T_{j_{n}}Lx_{n}-Lu,T_{j_{n}}Lx_{n}-Lx_{n} \rangle \\& \quad =- \Vert T_{j_{n}}Lx_{n}-Lx_{n} \Vert ^{2}+\langle T_{j_{n}}Lx_{n}-Lu,T_{j _{n}}Lx_{n}-Lx_{n} \rangle \\& \quad \leq - \Vert T_{j_{n}}Lx_{n}-Lx_{n} \Vert ^{2}+\frac{1}{2} \Vert T_{j_{n}}Lx_{n}-Lx _{n} \Vert ^{2} \\& \quad =-\frac{1}{2} \Vert T_{j_{n}}Lx_{n}-Lx_{n} \Vert ^{2}. \end{aligned}$$

It follows from (3.2) and (3.3) that

3.4 $$\begin{aligned} \Vert y_{n}-u \Vert ^{2} \leq & \Vert x_{n}-u \Vert ^{2}-\lambda_{n} \Vert T_{j_{n}}Lx_{n}-Lx _{n} \Vert ^{2}+ \lambda_{n}^{2} \Vert L \Vert ^{2} \Vert T_{j_{n}}Lx_{n}-Lx_{n} \Vert ^{2} \\ =& \Vert x_{n}-u \Vert ^{2}-\lambda_{n} \bigl(1-\lambda_{n} \Vert L \Vert ^{2}\bigr) \Vert T_{j_{n}}Lx _{n}-Lx_{n} \Vert ^{2} \\ \leq & \Vert x_{n}-u \Vert ^{2}. \end{aligned}$$

Hence, from Lemma 2.2, Lemma 2.4, and the control conditions on $\{\beta_{n}\}$ and $\{\lambda_{n}\}$, we have

$$\begin{aligned} \Vert x_{n+1}-u \Vert ^{2} =& \Vert z_{i_{n},n}-u \Vert ^{2} \\ =& \bigl\Vert J_{\beta_{n}}^{B_{i_{n}}}(I-\beta_{n} A_{i_{n}})y_{n}-J_{\beta _{n}}^{B_{i_{n}}}(I- \beta_{n} A_{i_{n}})u \bigr\Vert ^{2} \\ \leq & \bigl\Vert (I-\beta_{n} A_{i_{n}})y_{n}-(I- \beta_{n} A_{i_{n}})u \bigr\Vert ^{2} \\ =& \Vert y_{n}-u \Vert ^{2}+\beta_{n}^{2} \Vert A_{i_{n}}y_{n}- A_{i_{n}}u \Vert ^{2}-2 \beta_{n}\langle y_{n}-u, A_{i_{n}}y_{n}- A_{i_{n}}u\rangle \\ \leq & \Vert y_{n}-u \Vert ^{2}+\beta_{n}^{2} \Vert A_{i_{n}}y_{n}- A_{i_{n}}u \Vert ^{2}-2\beta_{n}\alpha_{i_{n}} \Vert A_{i_{n}}y_{n}- A_{i_{n}}u \Vert ^{2} \\ \leq & \Vert y_{n}-u \Vert ^{2}+\beta_{n}( \beta_{n}-2\alpha_{i_{n}}) \Vert A_{i_{n}}y _{n}- A_{i_{n}}u \Vert ^{2} \\ \leq & \Vert y_{n}-u \Vert ^{2} \\ \leq & \Vert x_{n}-u \Vert ^{2}. \end{aligned}$$

This means that $\|x_{n}-u\|$ is a nonincreasing sequence of nonnegative real numbers, so it follows that it is a convergent sequence. Also, from the above inequality, we have $\|x_{n}-u\|$ and $\|y_{n}-u\|$ converge to the same limit point. These imply that the sequences $\{x_{n}\}$ and $\{y_{n}\}$ are bounded, and the proof is completed. □

### Lemma 3.2

*If* $0< a\leq \lambda_{n}\leq b<\frac{1}{2\|L\|^{2}}$, *then* $\omega_{w}(Lx _{n})\subset \bigcap_{j=1}^{N}F(T_{j})$.

### Proof

By (3.4) we have

$$\begin{aligned} \lambda_{n}\bigl(1-\lambda_{n} \Vert L \Vert ^{2}\bigr) \Vert T_{j_{n}}Lx_{n}-Lx_{n} \Vert ^{2} \leq & \Vert x_{n}-u \Vert ^{2}- \Vert y_{n}-u \Vert ^{2}\rightarrow 0, \quad n\rightarrow \infty, \end{aligned}$$

and hence,

$$\begin{aligned} \Vert T_{j_{n}}Lx_{n}-Lx_{n} \Vert \rightarrow 0, \quad n\rightarrow \infty. \end{aligned}$$

Therefore, from (3.1), we get

3.5 $$\begin{aligned} \bigl\Vert L^{*}(T_{j}Lx_{n}-Lx_{n}) \bigr\Vert =&\frac{1}{\lambda_{n}} \Vert y_{j,n}-x_{n} \Vert \\ \leq& \frac{1}{\lambda_{n}} \Vert y_{n}-x_{n} \Vert \\ = &\bigl\Vert L^{\ast }(T_{j_{n}}Lx _{n}-Lx_{n}) \bigr\Vert \\ \leq & \Vert L \Vert \Vert T_{j_{n}}Lx_{n}-Lx_{n} \Vert \rightarrow 0, \quad n\rightarrow \infty, \end{aligned}$$

for each $j=1, \ldots, N$, which implies that

3.6 $$\begin{aligned} \bigl\Vert L^{*}(T_{j}Lx_{n}-Lx_{n}) \bigr\Vert \rightarrow 0, \quad n\rightarrow \infty. \end{aligned}$$

From (2.1), we have

3.7 $$\begin{aligned}& \bigl\langle \lambda_{n} L^{*}(T_{j}Lx_{n}-Lx_{n}) +x_{n}-u,-\lambda_{n} L^{*}(T_{j}Lx_{n}-Lx_{n}) \bigr\rangle \\& \quad = -\lambda_{n}^{2} \bigl\Vert L^{*}(T_{j}Lx_{n}-Lx_{n}) \bigr\Vert ^{2}-\lambda_{n} \langle Lx_{n}-Lu, T_{j}Lx_{n}-Lx_{n}\rangle \\& \quad = -\lambda_{n}^{2} \bigl\Vert L^{*}(T_{j}Lx_{n}-Lx_{n}) \bigr\Vert ^{2}-\lambda_{n} \langle Lx_{n}-T_{j}Lx_{n}+T_{j}Lx_{n}-Lu, T_{j}Lx_{n}-Lx_{n}\rangle \\& \quad = -\lambda_{n}^{2} \bigl\Vert L^{*}(T_{j}Lx_{n}-Lx_{n}) \bigr\Vert ^{2}+\lambda_{n} \Vert T _{j}Lx_{n}-Lx_{n} \Vert ^{2}-\lambda_{n}\langle T_{j}Lx_{n}-Lu, T_{j}Lx _{n}-Lx_{n}\rangle \\& \quad \geq -\lambda_{n}^{2} \Vert L \Vert ^{2} \Vert T_{j}Lx_{n}-Lx_{n} \Vert ^{2}+\lambda _{n} \Vert T_{j}Lx_{n}-Lx_{n} \Vert ^{2}-\frac{1}{2}\lambda_{n} \Vert T_{j}Lx_{n}-Lx _{n} \Vert \\& \quad =\lambda_{n}\biggl(\frac{1}{2} -\lambda_{n} \Vert L \Vert ^{2}\biggr) \Vert T_{j}Lx_{n}-Lx_{n} \Vert ^{2}\geq 0 \end{aligned}$$

for each $j=1, \ldots, N$. Thus, by (3.6) and the assumption of $\{\lambda_{n}\}$, we have

3.8 $$\begin{aligned} \Vert T_{j}Lx_{n}-Lx_{n} \Vert \rightarrow 0, \quad n\rightarrow \infty, \end{aligned}$$

for each $j=1, \ldots, N$. From Lemma 2.1, we obtain $\omega_{w}(Lx_{n})\subset F(T_{j})$ for each $j=1, \ldots, N$. This completes the proof. □

### Lemma 3.3

*Let* $\alpha =\min \{\alpha_{1}, \ldots,\alpha_{M}\}$. *If* $\{\beta _{n}\}\subset (0, 2\alpha)$. *Then*, *for each* $i=1, \ldots, M$, *we have* $\|x_{n}-z_{i,n}\|\rightarrow 0$.

### Proof

Since $J_{\beta_{n}}^{B_{i}}$ and $I-\beta_{n}A_{i}$ are firmly nonexpansive, they are both $\frac{1}{2}$-averaged and hence $T_{i,n}:=J_{\beta_{n}}^{B_{i}}(I-\beta_{n}A_{i})$ is $\frac{3}{4}$-averaged by Lemma 2.3. Thus, for each $n\in \mathbb{N}$ and $1\leq i\leq M$, we can write

$$\begin{aligned} T_{i,n} =\frac{1}{4}I+\frac{3}{4}S_{i,n}, \end{aligned}$$

where $S_{i,n}$ is a nonexpansive mapping and $F(S_{i,n})=F(T_{i,n})=F(J _{\beta_{n}}^{B_{i}}(I-\beta_{n}A_{i}))=(A_{i}+B_{i})^{-1}(0)$ for each $n\in \mathbb{N}$ and $1\leq i\leq M$. Then we can rewrite $x_{n+1}$ as

3.9 $$\begin{aligned} x_{n+1}=T_{i_{n},n}(y_{n})= \frac{1}{4}y_{n}+\frac{3}{4}S_{i_{n},n}(y _{n}). \end{aligned}$$

Let $u\in \bigcap_{i=1}^{M}(A_{i}+B_{i})^{-1}(0)$, we have

$$\begin{aligned} \Vert x_{n+1}-u \Vert ^{2} =& \biggl\Vert \frac{1}{4}(y_{n}-u)+\frac{3}{4}\bigl(S_{i_{n},n}(y _{n})-u\bigr) \biggr\Vert ^{2} \\ =&\frac{1}{4} \Vert y_{n}-u \Vert ^{2}+ \frac{3}{4} \bigl\Vert S_{i_{n},n}(y_{n})-u \bigr\Vert ^{2}- \frac{3}{16} \bigl\Vert y_{n}-S_{i_{n},n}(y_{n}) \bigr\Vert ^{2} \\ \leq & \Vert y_{n}-u \Vert ^{2}-\frac{3}{16} \bigl\Vert y_{n}-S_{i_{n},n}(y_{n}) \bigr\Vert ^{2}, \end{aligned}$$

and hence,

$$\begin{aligned} \frac{3}{16} \bigl\Vert y_{n}-S_{i_{n},n}(y_{n}) \bigr\Vert ^{2}\leq \Vert y_{n}-u \Vert ^{2}- \Vert x _{n+1}-u \Vert ^{2}. \end{aligned}$$

Then

$$\begin{aligned} \bigl\Vert y_{n}-S_{i_{n},n}(y_{n}) \bigr\Vert \rightarrow 0, \quad n\rightarrow \infty. \end{aligned}$$

From (3.9),

3.10 $$\begin{aligned} \Vert y_{n}-x_{n+1} \Vert = \frac{3}{4} \bigl\Vert y_{n}-S_{i_{n},n}(y_{n}) \bigr\Vert \rightarrow 0, \quad n\rightarrow \infty. \end{aligned}$$

By (3.5), we get

3.11 $$\begin{aligned} \Vert x_{n}-y_{n} \Vert = \Vert x_{n}-y_{j_{n},n} \Vert \rightarrow 0, \quad n\rightarrow \infty. \end{aligned}$$

Now, from (3.1), (3.10), and (3.11), we obtain

3.12 $$\begin{aligned} \Vert x_{n}-z_{i,n} \Vert \leq \Vert x_{n}-z_{i_{n},n} \Vert \leq \Vert x_{n}-y_{n} \Vert + \Vert y _{n}-x_{n+1} \Vert \rightarrow 0, \quad n\rightarrow \infty. \end{aligned}$$

 □

### Lemma 3.4

*Assume that* $\beta_{n}\rightarrow \beta $ *for some positive real number* *β*. *Then*, *for each* $i=1, \ldots, M$, *we have* $\|x_{n}-J_{ \beta }^{B_{i}}(I-\beta A_{i})x_{n}\|\rightarrow 0$, $n\rightarrow \infty$.

### Proof

Set $w_{i,n}=(I-\beta_{n} A_{i})y_{n}$, so $z_{i,n}=J_{\beta_{n}}^{B _{i}} w_{i,n}$. By Lemma 2.5, we have

3.13 $$\begin{aligned}& \bigl\Vert J_{\beta_{n}}^{B_{i}} (I- \beta_{n} A_{i})y_{n}-J_{\beta }^{B_{i}} (I-\beta_{n} A_{i})y_{n} \bigr\Vert \\& \quad = \bigl\Vert J_{\beta_{n}}^{B_{i}} w_{i,n}-J_{\beta }^{B_{i}} w_{i,n} \bigr\Vert \\& \quad = \biggl\Vert J_{\beta }^{B_{i}} \biggl( \frac{\beta }{\beta_{n}} w_{i,n}+\biggl(1-\frac{ \beta }{\beta_{n}}\biggr)J_{\beta_{n}}^{B_{i}} w_{i,n} \biggr) -J_{\beta } ^{B_{i}} w_{i,n} \biggr\Vert \\& \quad \leq \biggl\Vert \frac{\beta }{\beta_{n}} w_{i,n}+\biggl(1- \frac{\beta }{\beta_{n}}\biggr)J _{\beta_{n}}^{B_{i}} w_{i,n}- w_{i,n} \biggr\Vert \\& \quad = \biggl\vert 1-\frac{\beta }{\beta_{n}} \biggr\vert \bigl\Vert J_{\beta_{n}}^{B _{i}} w_{i,n}- w_{i,n} \bigr\Vert . \end{aligned}$$

On the other hand, we have

$$\begin{aligned} \bigl\Vert J_{\beta_{n}}^{B_{i}} w_{i,n}- w_{i,n} \bigr\Vert =& \Vert z_{i,n}- w_{i,n} \Vert \\ =& \Vert z_{i,n}-y_{n}+\beta_{n} A_{i}y_{n} \Vert \\ \leq & \Vert z_{i,n}-x_{n} \Vert + \Vert x_{n}-y_{n} \Vert +\beta_{n} \Vert A_{i}y_{n} \Vert . \end{aligned}$$

Since $A_{i}$ is inverse strongly monotone, $\{y_{n}\}$ is bounded, (3.11) and (3.12) we know that $\lbrace \|J_{\beta_{n}} ^{B_{i}} w_{i,n}- w_{i,n}\| \rbrace $ is bounded. It follows from $\beta_{n}\rightarrow \beta $ and (3.13) that

3.14 $$\begin{aligned} \bigl\Vert J_{\beta_{n}}^{B_{i}} (I- \beta_{n} A_{i})y_{n}-J_{\beta }^{B_{i}} (I- \beta_{n} A_{i})y_{n} \bigr\Vert \rightarrow 0, \quad n\rightarrow \infty. \end{aligned}$$

We also have

3.15 $$\begin{aligned}& \bigl\Vert J_{\beta }^{B_{i}} (I- \beta_{n} A_{i})y_{n}-J_{\beta }^{B_{i}} (I- \beta A_{i})x_{n} \bigr\Vert \\& \quad \leq \bigl\Vert (I-\beta_{n} A_{i})y_{n}-(I- \beta A_{i})x_{n} \bigr\Vert \\& \quad \leq \Vert y_{n}-x_{n} \Vert + \beta_{n} \Vert A_{i}y_{n}-A_{i}x_{n} \Vert + \Vert \beta _{n} A_{i}x_{n}-\beta A_{i}x_{n} \Vert \\& \quad \leq \Vert y_{n}-x_{n} \Vert +\frac{\beta_{n}}{\alpha } \Vert y_{n}-x_{n} \Vert + \vert \beta_{n}- \beta \vert \Vert A_{i}x_{n} \Vert \\& \quad \leq \biggl(1+\frac{\beta_{n}}{\alpha }\biggr) \Vert y_{n}-x_{n} \Vert + \vert \beta_{n}- \beta \vert \Vert A_{i}x_{n} \Vert \rightarrow 0, \quad n\rightarrow \infty. \end{aligned}$$

It follows form (3.12), (3.14), and (3.15) that

$$\begin{aligned}& \bigl\Vert x_{n}-J_{\beta }^{B_{i}}(I-\beta A_{i})x_{n} \bigr\Vert \\& \quad \leq \Vert x_{n}-z_{i,n} \Vert + \bigl\Vert J_{\beta_{n}}^{B_{i}} (I-\beta_{n} A_{i})y_{n}-J_{\beta }^{B_{i}} (I-\beta_{n} A_{i})y_{n} \bigr\Vert \\& \qquad {}+ \bigl\Vert J_{\beta }^{B_{i}} (I-\beta_{n} A_{i})y_{n}-J_{\beta }^{B_{i}} (I- \beta A_{i})x_{n} \bigr\Vert \rightarrow 0, \quad n\rightarrow \infty, \end{aligned}$$

for each $i=1, \ldots, M$. This completes the proof of the lemma. □

Now, the weak convergence of algorithm (3.1) is given by the following theorem.

### Theorem 3.5

*Let* $H_{1}$ *and* $H_{2}$ *be real Hilbert spaces*. *Let* $T_{j}:H_{2} \rightarrow H_{2}$, $j=1, \ldots, N$, *be nonexpansive mappings*, $L:H_{1}\rightarrow H_{2}$ *be a bounded linear operator*, $A_{i}:H_{1} \rightarrow H_{1}$, $i=1, \ldots, M$, *be* $\alpha_{i}$-*inverse strongly monotone operators*, *and* $B_{i}:H_{1}\rightarrow 2^{H_{1}}$, $i=1, \ldots, M$, *be maximal monotone operators such that* $\mathcal{F}= ( \bigcap_{i=1}^{M}(A_{i}+B_{i})^{-1}(0) ) \cap L ^{-1} ( \bigcap_{j=1}^{N}F(T_{j}) ) \neq \emptyset $. *Let* $\alpha =\min \{\alpha_{1}, \ldots,\alpha_{M}\}$, $\beta_{n}\in (0, 2 \alpha)$ *for each* $n\in \mathbb{N}$ *and* $0< a\leq \lambda_{n}\leq b<\frac{1}{2 \|L\|^{2}}$, *then the sequence* $\{x_{n}\}$ *generated by* (3.1) *converges weakly to a point* $p\in \mathcal{F}$.

### Proof

In Lemma 3.1, we show that $\lim_{n\rightarrow \infty }\|x_{n}-u\|$ exists for each $u\in \mathcal{F}$. From Lemmas 3.2 and 3.4 we imply that $\omega_{w}(x_{n})\subset \mathcal{F}$. Then it follows from Lemma 2.6 that $\{x_{n}\}$ converges weakly to a point $p\in \mathcal{F}$. □

Recall that for a subset *C* of *H*, a mapping $T:C\rightarrow C$ is said to be semi-compact if for any bounded sequence $\{x_{n}\}\subset C$ such that $\|x_{n} -Tx_{n}\|\rightarrow 0$ ($n\rightarrow \infty $), there exists a subsequence $\{x_{n_{j}}\}$ of $\{x_{n}\}$ such that $\{x_{n_{j}}\}$ converges strongly to $x \in C$.

Strong convergence of algorithm (3.1), under the concept of semi-compact assumption, is given by the following theorem.

### Theorem 3.6

*Let* $H_{1}$ *and* $H_{2}$ *be real Hilbert spaces*. *Let* $T_{j}:H_{2} \rightarrow H_{2}$, $j=1, \ldots, N$, *be nonexpansive mappings*, $L:H_{1}\rightarrow H_{2}$ *be a bounded linear operator*, $A_{i}:H_{1} \rightarrow H_{1}$, $i=1, \ldots, M$, *be* $\alpha_{i}$-*inverse strongly monotone operators*, *and* $B_{i}:H_{1}\rightarrow 2^{H_{1}}$, $i=1, \ldots, M$, *be maximal monotone operators such that* $\mathcal{F}= ( \bigcap_{i=1}^{M}(A_{i}+B_{i})^{-1}(0) ) \cap L ^{-1} ( \bigcap_{j=1}^{N}F(T_{j}) ) \neq \emptyset $. *Let* $\alpha =\min \{\alpha_{1}, \ldots,\alpha_{M}\}$, $\beta_{n}\in (0, 2 \alpha)$ *for each* $n\in \mathbb{N}$ *and* $0< a\leq \lambda_{n}\leq b<\frac{1}{2 \|L\|^{2}}$. *If at least one of the maps* $T_{j}$ *is semi*-*compact*, *then the sequence* $\{x_{n}\}$ *generated by* (3.1) *converges strongly to a point* $p\in \mathcal{F}$.

### Proof

Let $T_{j}$ be semi-compact for some fixed $j\in \{1, \ldots, N\}$. Since $\lim_{n\rightarrow \infty }\|T_{j}Lx_{n}-Lx_{n}\|=0$ by (4.7), there exists a subsequence $\{x_{n_{k}}\}$ of $\{x_{n}\}$ such that it converges strongly to *q*. Since $\{x_{n}\}$ converges weakly to *p*, we get $p=q$. On the other hand, $\lim_{n\rightarrow \infty }\|x_{n}-p\|$ exists and $\lim_{n\rightarrow \infty }\|x_{n_{k}}-p\|=0$, which show that $\{x_{n}\}$ converges strongly to $p\in \mathcal{F}$. This completes the proof of the theorem. □

### Deduced results of parallel algorithm

One can obtain some results from Theorem 3.5. We give some of them in the following.

If we take $M=N=1$, we have the following corollary.

#### Corollary 3.7

*Let* $H_{1}$ *and* $H_{2}$ *be real Hilbert spaces*. *Let* $T:H_{2}\rightarrow H_{2}$ *be a nonexpansive mapping*, $L:H_{1}\rightarrow H_{2}$ *be a bounded linear operator*, $A:H_{1}\rightarrow H_{1}$ *be an* *α*-*inverse strongly monotone operator*, *and* $B:H_{1}\rightarrow 2^{H_{1}}$ *be a maximal monotone operator such that* $(A+B)^{-1}(0) \cap L^{-1}(F(T))\neq \emptyset $. *Suppose that the sequence* $\{x_{n}\}$ *is defined by the following algorithm*:

$$\begin{aligned} \textstyle\begin{cases} y_{n}=x_{n}+\lambda_{n} L^{*}( T-I)Lx_{n}, \\ x_{n+1}=J_{\beta }^{B}(I-\beta_{n} A)y_{n}, \end{cases}\displaystyle \end{aligned}$$

*where* $x_{1}\in H_{1}$, $0< a\leq \lambda_{n}\leq b<\frac{1}{2\|L\| ^{2}}$, *and* $\beta_{n}\in (0, 2\alpha)$ *for each* $n\in \mathbb{N}$. *Then the sequence* $\{x_{n}\}$ *converges weakly to a point* $p\in (A+B)^{-1}(0) \cap L^{-1}(F(T))$. *If* *T* *be semi*-*compact*, *then the convergence is strong*.

From Theorem 3.5, we have the following corollary for the problem of finding a common zero of the sum of *α*-inverse strongly monotone operators and maximal monotone operators.

#### Corollary 3.8

*Let* *H* *be a real Hilbert space*, $A_{i}:H\rightarrow H$, $i=1, \ldots, M$, *be* $\alpha_{i}$-*inverse strongly monotone operators*, *and* $B_{i}:H\rightarrow 2^{H}$, $i=1, \ldots, M$, *be maximal monotone operators such that* $\mathcal{F}=\bigcap_{i=1}^{M}(A_{i}+B_{i})^{-1}(0) \neq \emptyset $ *and* $\alpha =\min \{\alpha_{1}, \ldots,\alpha_{M}\}$. *Suppose that the sequence* $\{x_{n}\}$ *is defined by the following algorithm*:

$$\begin{aligned} \textstyle\begin{cases} z_{i,n}=J_{\beta_{n}}^{B_{i}}(I-\beta_{n} A_{i})x_{n}, \quad i=1, \ldots, M, \\ \textit{choose} \quad i_{n}: \Vert z_{i_{n},n}-x_{n} \Vert =\max_{i=1, \ldots, M} \Vert z_{i,n}-x _{n} \Vert , \\ x_{n+1}=z_{i_{n},n}, \end{cases}\displaystyle \end{aligned}$$

*where* $x_{1}\in H$ *and* $\beta_{n}\in (0, 2\alpha)$ *for each* $n\in \mathbb{N}$. *Then the sequence* $\{x_{n}\}$ *converges weakly to a point* $p\in \bigcap_{i=1}^{M}(A_{i}+B_{i})^{-1}(0)$.

In the following corollary, we have a result for finding a common zero of a finite family of maximal monotone operators.

#### Corollary 3.9

*Let* *H* *be a real Hilbert space*, $B_{i}:H\rightarrow 2^{H}$, $i=1, \ldots, M$, *be maximal monotone operators such that* $\bigcap_{i=1} ^{M}B_{i}^{-1}(0)\neq \emptyset $. *Suppose that the sequence* $\{x_{n}\}$ *is defined by the following algorithm*:

$$\begin{aligned} \textstyle\begin{cases} z_{i,n}=J_{\beta_{n}}^{B_{i}}x_{n}, \quad i=1, \ldots, M, \\ \textit{choose}\quad i_{n}: \Vert z_{i_{n},n}-x_{n} \Vert =\max_{i=1, \ldots, M} \Vert z_{i,n}-x _{n} \Vert , \\ x_{n+1}=z_{i_{n},n}, \end{cases}\displaystyle \end{aligned}$$

*where* $x_{1}\in H$ *and* $\beta_{n}>0$ *for each* $n\in \mathbb{N}$. *Then the sequence* $\{x_{n}\}$ *converges weakly to a point* $p\in \bigcap_{i=1} ^{M}B_{i}^{-1}(0)$.

#### Corollary 3.10

*Let* *H* *be a real Hilbert space*, $A_{i}:H\rightarrow H$, $i=1, \ldots, M$, *be* $\alpha_{i}$-*inverse strongly monotone operators such that* $\bigcap_{i=1}^{M}A_{i}^{-1}(0)\neq \emptyset $ *and* $\alpha =\min \{ \alpha_{1}, \ldots,\alpha_{M}\}$. *Suppose that the sequence* $\{x_{n}\}$ *is defined by the following algorithm*:

$$\begin{aligned} \textstyle\begin{cases} z_{i,n}=x_{n}-\beta_{n} A_{i} x_{n}, \quad i=1, \ldots, M, \\ \textit{choose}\quad i_{n}: \Vert z_{i_{n},n}-x_{n} \Vert =\max_{i=1, \ldots, M} \Vert z_{i,n}-x _{n} \Vert , \\ x_{n+1}=z_{i_{n},n}, \end{cases}\displaystyle \end{aligned}$$

*where* $x_{1}\in H$ *and* $\beta_{n}\in (0, 2\alpha)$ *for each* $n\in \mathbb{N}$. *Then the sequence* $\{x_{n}\}$ *converges weakly to a point* $p\in \bigcap_{i=1}^{M}A_{i}^{-1}(0)$.

#### Corollary 3.11

*Let* $H_{1}$ *and* $H_{2}$ *be real Hilbert spaces and* $T_{j}:H_{2}\rightarrow H_{2}$, $j=1, \ldots, N$, *be nonexpansive mappings and* $L:H_{1} \rightarrow H_{2}$ *be a bounded linear operator such that* $\mathcal{F}= L^{-1} ( \bigcap_{j=1}^{N}F(T_{j}) ) \neq \emptyset $. *Suppose that the sequence* $\{x_{n}\}$ *is defined by the following algorithm*:

$$\begin{aligned} \textstyle\begin{cases} y_{j,n}=x_{n}+\lambda_{n} L^{*}( T_{j}-I)Lx_{n}, \quad j=1, \ldots, N, \\ \textit{choose} \quad j_{n}: \Vert y_{j_{n},n}-x_{n} \Vert =\max_{j=1, \ldots, N} \Vert y_{j,n}-x _{n} \Vert , \\ x_{n+1}=y_{j_{n},n}, \end{cases}\displaystyle \end{aligned}$$

*where* $x_{1}\in H$ *and* $0< a\leq \lambda_{n}\leq b< \frac{1}{2\|L\|^{2}}$. *Then the sequence* $\{x_{n}\}$ *converges weakly to a point* $p\in \bigcap_{j=1}^{N}F(T_{j})$. *If* $T_{j}$ *is semi*-*compact for some* $1\leq j \leq N$, *then the convergence is strong*.

## Parallel hybrid algorithm

Notice that, in order to guarantee the strong convergence theorem of the introduced algorithm (3.1), we proposed an additional assumption to one of the operators $T_{j}$, as a semi-compact assumption (see Theorem 3.6). Next, we propose the following hybrid algorithm to obtain a strong convergence theorem for finding a point in zeros of a finite family of sums of *α*-inverse strongly monotone operators and maximal monotone operators and nonexpansive mappings. Of course, the strong convergence theorems of the following algorithm will be guaranteed without any additional assumptions on the considered operators. To do this, we recall some necessary concepts and facts: let *C* be a closed and convex subset of a Hilbert space *H*. The operator $P_{C}$ is called a metric projection operator if it assigns to each $x\in H$ its nearest point $y\in C$ such that

$$\begin{aligned} \Vert x-y \Vert = \min \bigl\{ \Vert x-z \Vert : z \in C\bigr\} . \end{aligned}$$

An element *y* is called the metric projection of *H* onto *C* and is denoted by $P_{C}x$. It exists and is unique at any point of the Hilbert space. It is known that the metric projection operator $P_{C}$ is a firmly nonexpansive mapping. Also, the following characterization is very useful in our proof.

### Lemma 4.1

*Let* *H* *be a Hilbert space and* *C* *be a nonempty*, *closed*, *and convex subset of* *H*. *Then*, *for all* $x\in H$, *the element* $z=P_{C}x$ *if and only if*

$$\begin{aligned} \langle x-z, z-y\rangle \geq 0, \quad \forall y\in C. \end{aligned}$$

Now we are in a position to introduce the aforementioned algorithm: Let $x_{1}\in C_{1}=H_{1}$ and $\{x_{n}\}$ be a sequence generated by the following algorithm:

4.1 $$\begin{aligned} \textstyle\begin{cases} y_{j,n}=x_{n}+\lambda_{n} L^{*}( T_{j}-I)Lx_{n},\quad j=1, \ldots, N, \\ \mbox{choose}\quad j_{n}: \Vert y_{j_{n},n}-x_{n} \Vert =\max_{j=1, \ldots, N} \Vert y_{j,n}-x _{n} \Vert , \\ y_{n}=y_{j_{n},n}, \\ z_{i,n}=J_{\beta_{n}}^{B_{i}}(I-\beta_{n} A_{i})y_{n},\quad i=1, \ldots, M, \\ \mbox{choose}\quad i_{n}: \Vert z_{i_{n},n}-x_{n} \Vert =\max_{i=1, \ldots, M} \Vert z_{i,n}-x _{n} \Vert , \\ z_{n}=z_{i_{n},n}, \\ C_{n+1}=\{ z\in C_{n}: \Vert z_{n}-z \Vert \leq \Vert y_{n}-z \Vert \leq \Vert x_{n}-z \Vert \}, \\ x_{n+1}=P_{C_{n+1}}x_{1}. \end{cases}\displaystyle \end{aligned}$$

### Theorem 4.2

*Let* $H_{1}$ *and* $H_{2}$ *be real Hilbert spaces*. *Let* $T_{j}:H_{2} \rightarrow H_{2}$, $j=1, \ldots, N$, *be nonexpansive mappings*, $L:H_{1}\rightarrow H_{2}$ *be a bounded linear operator*, $A_{i}:H_{1} \rightarrow H_{1}$, $i=1, \ldots, M$, *be* $\alpha_{i}$-*inverse strongly monotone operators*, *and* $B_{i}:H_{1}\rightarrow 2^{H_{1}}$, $i=1, \ldots, M$, *be maximal monotone operators such that* $\mathcal{F}= ( \bigcap_{i=1}^{M}(A_{i}+B_{i})^{-1}(0) ) \cap L ^{-1} ( \bigcap_{j=1}^{N}F(T_{j}) ) \neq \emptyset $. *Let* $\alpha =\min \{\alpha_{1}, \ldots,\alpha_{M}\}$, $\beta_{n}\in (0, 2 \alpha)$ *for each* $n\in \mathbb{N}$ *and* $0< a\leq \lambda_{n}\leq b<\frac{1}{2 \|L\|^{2}}$. *Then the sequence* $\{x_{n}\}$ *generated by* (4.1) *converges strongly to* $q=P_{\mathcal{F}}(x_{1})$.

### Proof

We prove that the sequence $\{x_{n}\}$ generated by (4.1) is well defined. We first show that $C_{n}$ is closed and convex for each $n\in \mathbb{N}$. $C_{1}=H_{1}$ is closed and convex and suppose that $C_{n}$ is closed and convex for some $n>1$. Set

$$\begin{aligned}& C_{n}^{1}=\bigl\{ z\in H_{1}: \Vert z_{n}-z \Vert \leq \Vert y_{n}-z \Vert \bigr\} , \\& C_{n}^{2}=\bigl\{ z\in H_{1}: \Vert y_{n}-z \Vert \leq \Vert x_{n}-z \Vert \bigr\} , \end{aligned}$$

then $C_{n+1}=C_{n}\cap C_{n}^{1}\cap C_{n}^{2}$. For each $p\in C _{n}^{1}$, we obtain

$$\begin{aligned}& \Vert z_{n}-p \Vert \leq \Vert y_{n}-p \Vert \\& \quad \Longleftrightarrow \quad \Vert z_{n}-y_{n}+y_{n}-p \Vert ^{2}\leq \Vert y_{n}-p \Vert ^{2} \\& \quad \Longleftrightarrow \quad \Vert z_{n}-y_{n} \Vert ^{2}+ \Vert y_{n}-p \Vert ^{2}+2\langle z _{n}-y_{n},y_{n}-p\rangle \leq \Vert y_{n}-p \Vert ^{2} \\& \quad \Longleftrightarrow \quad \Vert z_{n}-y_{n} \Vert ^{2}+2\langle z_{n}-y_{n},y_{n}-p \rangle \leq 0. \end{aligned}$$

This implies that $C_{n}^{1}$ is closed and convex. In a similar manner, $C_{n}^{2}$ is closed and convex and so is $C_{n+1}=C_{n}\cap C_{n} ^{1}\cap C_{n}^{2}$. By the induction, $C_{n}$ is closed and convex for each $n\geq 1$.

We show that $\mathcal{F}\subset C_{n}$ for each $n\geq 1$. Let $p\in \mathcal{F}$. From Lemmas 2.2 and 2.4 and (4.1), we have

$$\begin{aligned} \Vert z_{n}-p \Vert =& \bigl\Vert J_{\beta_{n}}^{B_{i_{n}}}(I-\beta_{n} A_{i_{n}})y_{n}-J_{\beta _{n}}^{B_{i_{n}}}(I- \beta_{n} A_{i_{n}})p \bigr\Vert \\ \leq & \bigl\Vert (I-\beta_{n} A_{i_{n}})y_{n}-(I- \beta_{n} A_{i_{n}})p \bigr\Vert \\ \leq & \Vert y_{n}-p \Vert . \end{aligned}$$

This together with (3.4) implies that $p\in C_{n+1}$. Then $\{x_{n}\}$ is well defined.

Since $\mathcal{F}$ is nonempty, closed, and convex, there exists a unique element $q\in \mathcal{F}\subset C_{n}$ such that $q=P_{ \mathcal{F}}x_{1}$. From $x_{n+1}=P_{C_{n+1}} (x_{1})$, we get

4.2 $$\begin{aligned} \Vert x_{n+1}-x_{1} \Vert \leq \Vert x_{1}-q \Vert . \end{aligned}$$

Since again $x_{n}=P_{C_{n}} (x_{1})$ and $x_{n+1}=P_{C_{n+1}} (x_{1}) \in C_{n+1}\subset D_{n} $, we get

4.3 $$\begin{aligned} \Vert x_{n}-x_{1} \Vert \leq \Vert x_{n+1}-x_{1} \Vert . \end{aligned}$$

Thus, the sequence $\{\|x_{n}-x_{1}\|\}$ is a bounded above and nondecreasing sequence, so $\lim_{n\rightarrow \infty } \|x _{n}-x_{1}\|$ exists, and the sequence $\{x_{n}\}$ is bounded. By (3.4) the sequence $\{y_{n}\}$ is bounded too.

We show that $\|x_{n+1}-x_{n}\|\rightarrow 0$, $\|x_{n}-y_{n}\|\rightarrow 0$, and $\|y_{n}-z_{n}\|\rightarrow 0$. From $x_{n}=P_{C_{n}} (x_{1})$, $x_{n+1}=P_{C_{n+1}} (x_{1})\in C_{n+1}\subset C_{n} $, and Lemma 4.1, we obtain

$$\begin{aligned} \langle x_{1}-x_{n}, x_{n}-x_{n+1} \rangle \geq 0. \end{aligned}$$

Then we get

$$\begin{aligned}& \Vert x_{n}-x_{n+1} \Vert ^{2} \\& \quad = \Vert x_{n}-x_{1}+x_{1}-x_{n+1} \Vert ^{2} \\& \quad = \Vert x_{n}-x_{1} \Vert ^{2}+2 \langle x_{n}-x_{1},x_{1}-x_{n+1}\rangle + \Vert x _{1}-x_{n+1} \Vert ^{2} \\& \quad = \Vert x_{n}-x_{1} \Vert ^{2}+2 \langle x_{n}-x_{1},x_{1}-x_{n}\rangle +2 \langle x_{n}-x_{1},x_{n}-x_{n+1} \rangle + \Vert x_{1}-x_{n+1} \Vert ^{2} \\& \quad \leq \Vert x_{n}-x_{1} \Vert ^{2}-2 \langle x_{n}-x_{1},x_{n}-x_{1}\rangle + \Vert x_{1}-x_{n+1} \Vert ^{2} \\& \quad = \Vert x_{n}-x_{1} \Vert ^{2}-2 \Vert x_{n}-x_{1} \Vert ^{2}+ \Vert x_{1}-x_{n+1} \Vert ^{2} \\& \quad =- \Vert x_{n}-x_{1} \Vert ^{2}+ \Vert x_{1}-x_{n+1} \Vert ^{2}\rightarrow 0, \quad n\rightarrow \infty, \end{aligned}$$

and hence,

$$\begin{aligned} \Vert x_{n}-x_{n+1} \Vert \rightarrow 0, \quad n \rightarrow \infty. \end{aligned}$$

By $x_{n+1}=P_{C_{n+1}} (x_{1})\in C_{n+1}\subset C_{n} $ and the definition of $C_{n}$, we obtain

$$\begin{aligned} \Vert x_{n+1}-z_{n} \Vert \leq \Vert x_{n+1}-y_{n} \Vert \leq \Vert x_{n+1}-x_{n} \Vert , \end{aligned}$$

and then

$$\begin{aligned} \Vert x_{n}-y_{n} \Vert \leq \Vert x_{n}-x_{n+1} \Vert + \Vert x_{n+1}-y_{n} \Vert \leq 2 \Vert x_{n}-x _{n+1} \Vert , \end{aligned}$$

which implies that

4.4 $$\begin{aligned} \Vert x_{n}-y_{n} \Vert \rightarrow 0, \quad n\rightarrow \infty. \end{aligned}$$

Also, we have

$$\begin{aligned} \Vert y_{n}-z_{n} \Vert \leq& \Vert y_{n}-x_{n+1} \Vert + \Vert x_{n+1}-z_{n} \Vert \\ \leq& 2 \Vert x_{n}-x _{n+1} \Vert , \end{aligned}$$

therefore,

4.5 $$\begin{aligned} \Vert y_{n}-z_{n} \Vert \rightarrow 0, \quad n\rightarrow \infty. \end{aligned}$$

By (4.4) and (4.5), we obtain

4.6 $$\begin{aligned} \Vert x_{n}-z_{n} \Vert \rightarrow 0, \quad n\rightarrow \infty. \end{aligned}$$

Now, we show that $\omega_{w}(x_{n})\subset \mathcal{F}$. From (3.5), (3.7), and (4.4), we get

4.7 $$\begin{aligned} \Vert T_{j}Lx_{n}-Lx_{n} \Vert \rightarrow 0, \quad n\rightarrow \infty, \end{aligned}$$

for each $j=1, \ldots, N$. It follows from Lemma 2.1 that $\omega_{w}(Lx_{n})\subset \bigcap_{j=1}^{N}F(T_{j})$. By arguing similarly to the proof of Lemma 3.4, (4.4), and (4.6), we conclude $\omega_{w}(x_{n})\subset F(J_{\beta }^{B_{i}}(I-\beta A_{i}))= \bigcap_{i=1}^{M}(A_{i}+B_{i})^{-1}(0)$. Therefore,

4.8 $$\begin{aligned} \omega_{w}(x_{n})\subset \mathcal{F}. \end{aligned}$$

Finally, we show that the sequence $\{x_{n}\}$ generated by (4.1) converges strongly to $q=P_{\mathcal{F}}(x_{1})$. Since $x_{n}=P_{C_{n}} (x_{1})$ and $q \in \mathcal{F}\subset C_{n}$, we get

4.9 $$\begin{aligned} \Vert x_{n}-x_{1} \Vert \leq \Vert q-x_{1} \Vert . \end{aligned}$$

Let $\{x_{n_{k}}\}$ be an arbitrary subsequence of $\{x_{n}\}$ converging weakly to $p\in H_{1}$. Then $p\in \mathcal{F}$ by (4.8) and hence it follows from the lower semi-continuity of the norm that

$$\begin{aligned} \Vert q-x_{1} \Vert \leq& \Vert p-x_{1} \Vert \\ \leq& \liminf_{k\rightarrow \infty } \Vert x_{n_{k}}-x_{1} \Vert \\ \leq& \limsup_{k\rightarrow \infty } \Vert x _{n_{k}}-x_{1} \Vert \\ \leq& \Vert q-x_{1} \Vert . \end{aligned}$$

Thus, we obtain that $\lim_{k\rightarrow \infty } \|x_{n_{k}}-x _{1}\|=\|p-x_{1}\|=\|q-x_{1}\|$. Using the Kadec–Klee property of $H_{1}$, we get that $\lim_{k\rightarrow \infty } x_{n_{k}}=p=q$. Since $\{x_{n_{k}}\}$ is an arbitrary weakly convergent subsequence of $\{x_{n}\}$ and $\lim_{n\rightarrow \infty } \|x_{n}-x_{1}\|$ exists, we can imply that $\{x_{n}\}$ converges strongly to *q*. This completes the proof. □

### Deduced results of the parallel hybrid algorithm

One can obtain some results from Theorem 4.2. We give some of them in the following.

If we take $M=N=1$, we have the following corollary.

#### Corollary 4.3

*Let* $H_{1}$ *and* $H_{2}$ *be real Hilbert spaces*. *Let* $T:H_{2}\rightarrow H_{2}$ *be a nonexpansive mapping*, $L:H_{1}\rightarrow H_{2}$ *be a bounded linear operator*, $A:H_{1}\rightarrow H_{1}$ *be an* $\alpha_{i}$-*inverse strongly monotone operator*, *and* $B:H_{1}\rightarrow 2^{H_{1}}$ *be a maximal monotone operator such that* $\mathcal{F}=(A+B)^{-1}(0) \cap L^{-1}(F(T))\neq \emptyset $. *Suppose that the sequence* $\{x_{n}\}$ *is defined by the following algorithm*:

$$\begin{aligned} \textstyle\begin{cases} y_{n}=x_{n}+\lambda_{n} L^{*}( T-I)Lx_{n}, \\ z_{n}=J_{\beta_{n}}^{B}(I-\beta_{n} A)y_{n}, \\ C_{n+1}=\{ z\in C_{n}: \Vert z_{n}-z \Vert \leq \Vert y_{n}-z \Vert \leq \Vert x_{n}-z \Vert \}, \\ x_{n+1}=P_{C_{n+1}}x_{1}, \end{cases}\displaystyle \end{aligned}$$

*where* $x_{1}\in C_{1}=H_{1}$, $0< a\leq \lambda_{n}\leq b<\frac{1}{2\|L \|^{2}}$, *and* $\beta_{n}\in (0, 2\alpha)$ *for each* $n\in \mathbb{N}$. *Then the sequence* $\{x_{n}\}$ *converges strongly to* $q=P_{\mathcal{F}}(x _{1})$.

From Theorem 4.2, we have the following corollary for the problem of finding a common zero of the sum of *α*-inverse strongly monotone operators and maximal monotone operators.

#### Corollary 4.4

*Let* *H* *be a real Hilbert space*, $A_{i}:H\rightarrow H$, $i=1, \ldots, M$, *be* $\alpha_{i}$-*inverse strongly monotone operators*, *and* $B_{i}:H\rightarrow 2^{H}$, $i=1, \ldots, M$, *be maximal monotone operators such that* $\mathcal{F}=\bigcap_{i=1}^{M}(A_{i}+B_{i})^{-1}(0) \neq \emptyset $ *and* $\alpha =\min \{\alpha_{1}, \ldots,\alpha_{M}\}$. *Suppose that the sequence* $\{x_{n}\}$ *is defined by the following algorithm*:

$$\begin{aligned} \textstyle\begin{cases} z_{i,n}=J_{\beta_{n}}^{B_{i}}(I-\beta_{n} A_{i})x_{n}, \quad i=1, \ldots, M, \\ \textit{choose} \quad i_{n}: \Vert z_{i_{n},n}-x_{n} \Vert =\max_{i=1, \ldots, M} \Vert z_{i,n}-x _{n} \Vert , \\ z_{n}=z_{i_{n},n}, \\ C_{n+1}=\{ z\in C_{n}: \Vert z_{n}-z \Vert \leq \Vert x_{n}-z \Vert \}, \\ x_{n+1}=P_{C_{n+1}}x_{1}, \end{cases}\displaystyle \end{aligned}$$

*where* $x_{1}\in H$ *and* $\beta_{n}\in (0, 2\alpha)$ *for each* $n\in \mathbb{N}$. *Then the sequence* $\{x_{n}\}$ *converges strongly to* $q=P_{\mathcal{F}}(x_{1})$.

## Applications

### Zeros of maximal monotone operators

In this section, we discuss some applications of the main theorems. Let $M_{j}:H_{2}\rightarrow 2^{H_{2}}$, $j=1, \ldots, N$, be maximal monotone operators. Set $T_{j}=J_{r}^{M_{j}}$, where $r>0$ and $j=1, \ldots, N$. We know that $T_{j}$ is nonexpansive and $F(T_{j})=M_{j}^{-1}(0)$ for each $j=1, \ldots, N$. By applying Theorem 3.5, we can get the following results.

#### Theorem 5.1

*Let* $H_{1}$ *and* $H_{2}$ *be real Hilbert spaces*, $A_{i}:H_{1}\rightarrow H_{1}$, $i=1, \ldots, M$, *be* $\alpha_{i}$-*inverse strongly monotone operators*, $B_{i}:H_{1}\rightarrow 2^{H_{1}}$, $i=1, \ldots, M$, *and* $M_{j}:H_{2}\rightarrow 2^{H_{2}}$, $j=1, \ldots, N$, *be maximal monotone operators*, *and* $L:H_{1}\rightarrow H_{2}$ *be a bounded linear operator such that* $\mathcal{F}= ( \bigcap_{i=1}^{M}(A_{i}+B_{i})^{-1}(0) ) \cap L^{-1} ( \bigcap_{j=1}^{N}M_{j}^{-1}(0) ) \neq \emptyset $. *Let* $x_{1}\in H_{1}$ *and the sequence* $\{x_{n}\}$ *be generated by the following algorithm*:

$$\begin{aligned} \textstyle\begin{cases} y_{j,n}=x_{n}+\lambda_{n} L^{*}( J_{r}^{M_{j}}-I)Lx_{n}, \quad j=1, \ldots, N, \\ \textit{choose} \quad j_{n}: \Vert y_{j_{n},n}-x_{n} \Vert =\max_{j=1, \ldots, N} \Vert y_{j,n}-x _{n} \Vert , \\ y_{n}=y_{j_{n},n}, \\ z_{i,n}=J_{\beta_{n}}^{B_{i}}(I-\beta_{n} A_{i})y_{n}, \quad i=1, \ldots, M, \\ \textit{choose} \quad i_{n}: \Vert z_{i_{n},n}-x_{n} \Vert =\max_{i=1, \ldots, M} \Vert z_{i,n}-x _{n} \Vert , \\ x_{n+1}=z_{i_{n},n}. \end{cases}\displaystyle \end{aligned}$$

*If* $\alpha =\min \{\alpha_{1}, \ldots,\alpha_{M}\}$, $\beta_{n} \in (0, 2\alpha)$, *and* $0< a\leq \lambda_{n}\leq b<\frac{1}{2\|L\| ^{2}}$ *for each* $n\in \mathbb{N}$, *then* $\{x_{n}\}$ *converges weakly to a point* $p\in \mathcal{F}$.

By Theorem 5.1, we have the following corollary for multiple sets split null point problems.

#### Corollary 5.2

*Let* $H_{1}$ *and* $H_{2}$ *be real Hilbert spaces*, $B_{i}:H_{1}\rightarrow 2^{H_{1}}$, $i=1, \ldots, M$, $M_{j}:H_{2}\rightarrow 2^{H_{2}}$, $j=1, \ldots, N$, *be maximal monotone operators*, *and* $L:H_{1}\rightarrow H_{2}$ *be a bounded linear operator such that* $( \bigcap_{i=1}^{M}B _{i}^{-1}(0) ) \cap L^{-1} ( \bigcap_{j=1}^{N}M_{j}^{-1}(0) ) \neq \emptyset $. *Let* $x_{1}\in H_{1}$ *and the sequence* $\{x_{n}\}$ *be generated by the following algorithm*:

$$\begin{aligned} \textstyle\begin{cases} y_{j,n}=x_{n}+\lambda_{n} L^{*}( J_{r}^{M_{j}}-I)Lx_{n}, \quad j=1, \ldots, N, \\ \textit{choose} \quad j_{n}: \Vert y_{j_{n},n}-x_{n} \Vert =\max_{j=1, \ldots, N} \Vert y_{j,n}-x _{n} \Vert , \\ y_{n}=y_{j_{n},n}, \\ z_{i,n}=J_{\beta_{n}}^{B_{i}}y_{n}, \quad i=1, \ldots, M, \\ \textit{choose} \quad i_{n}: \Vert z_{i_{n},n}-x_{n} \Vert =\max_{i=1, \ldots, M} \Vert z_{i,n}-x _{n} \Vert , \\ x_{n+1}=z_{i_{n},n}. \end{cases}\displaystyle \end{aligned}$$

*If* $\beta_{n}>0$ *and* $0< a\leq \lambda_{n}\leq b<\frac{1}{2\|L\|^{2}}$ *for each* $n\in \mathbb{N}$, *then* $\{x_{n}\}$ *converges weakly to a point* $p\in ( \bigcap_{i=1}^{M}B_{i}^{-1}(0) ) \cap L^{-1} ( \bigcap_{j=1}^{N}M_{j}^{-1}(0) ) $.

By applying Theorem 4.2, we have the following theorem.

#### Theorem 5.3

*Let* $H_{1}$ *and* $H_{2}$ *be real Hilbert spaces*, $A_{i}:H_{1}\rightarrow H_{1}$, $i=1, \ldots, M$, *be* $\alpha_{i}$-*inverse strongly monotone operators*, $B_{i}:H_{1}\rightarrow 2^{H_{1}}$, $i=1, \ldots, M$, *and* $M_{j}:H_{2}\rightarrow 2^{H_{2}}$, $j=1, \ldots, N$, *be maximal monotone operators*, *and* $L:H_{1}\rightarrow H_{2}$ *be a bounded linear operator such that* $\mathcal{F}= ( \bigcap_{i=1}^{M}(A_{i}+B_{i})^{-1}(0) ) \cap L^{-1} ( \bigcap_{j=1}^{N}M_{j}^{-1}(0) ) \neq \emptyset $. *Let* $x_{1}\in H_{1}$ *and the sequence* $\{x_{n}\}$ *be generated by the following algorithm*:

5.1 $$\begin{aligned} \textstyle\begin{cases} y_{j,n}=x_{n}+\lambda_{n} L^{*}( J_{r}^{M_{j}}-I)Lx_{n}, \quad j=1, \ldots, N, \\ \textit{choose} \quad j_{n}: \Vert y_{j_{n},n}-x_{n} \Vert =\max_{j=1, \ldots, N} \Vert y_{j,n}-x _{n} \Vert , \\ y_{n}=y_{j_{n},n}, \\ z_{i,n}=J_{\beta_{n}}^{B_{i}}(I-\beta_{n} A_{i})y_{n}, \quad i=1, \ldots, M, \\ \textit{choose} \quad i_{n}: \Vert z_{i_{n},n}-x_{n} \Vert =\max_{i=1, \ldots, M} \Vert z_{i,n}-x _{n} \Vert , \\ z_{n}=z_{i_{n},n}, \\ C_{n+1}=\{ z\in C_{n}: \Vert z_{n}-z \Vert \leq \Vert y_{n}-z \Vert \leq \Vert x_{n}-z \Vert \}, \\ x_{n+1}=P_{C_{n+1}}x_{1}. \end{cases}\displaystyle \end{aligned}$$

*If* $\alpha =\min \{\alpha_{1}, \ldots,\alpha_{M}\}$, $\beta_{n} \in (0, 2\alpha)$, *and* $0< a\leq \lambda_{n}\leq b<\frac{1}{2\|L\| ^{2}}$ *for each* $n\in \mathbb{N}$, *then* $\{x_{n}\}$ *converges strongly to* $q=P_{\mathcal{F}}(x_{1})$.

### Multiple set split convex feasibility problems

Let $f\colon H\rightarrow \mathbb{R}\cup \{+\infty \}$ be a proper, convex, and lower semi-continuous function. It is well known that the subdifferential $\partial f\colon H \rightarrow 2^{H}$, which is defined as

$$ \partial f(x)= \bigl\{ z \in H : \langle y-x, z \rangle \leq f(y)-f(x), \forall y \in H\bigr\} , $$

is a maximal monotone operator. In particular, let *C* be a nonempty, closed, and convex subset of a real Hilbert space *H*. Let us consider the indicator function of *C*, denoted by $\iota_{C}$, which is defined as

$$\begin{aligned} \iota_{C}(x)=\textstyle\begin{cases} 0, & x\in C, \\ +\infty, & x\notin C. \end{cases}\displaystyle \end{aligned}$$

We know that $\iota_{C}$ is a proper, convex, and lower semi-continuous function on *H*, and it follows that the subdifferential $\partial \iota_{C}$ of $\iota_{C}$ is a maximal monotone operator. Furthermore, we get $z=J_{r}^{\partial \iota_{C}}x$ if and only if $z=P_{C}(x)$, where $x\in H$ and $J_{r}^{\partial \iota_{C}}=(I+r\partial \iota_{C})^{-1}$ for each $r>0$. Using these facts, by Theorems 3.5 and 4.2, we have the following corollaries for the multiple set split convex feasibility problem in Hilbert spaces.

#### Corollary 5.4

*Let* $H_{1}$ *and* $H_{2}$ *be real Hilbert spaces*, $C_{i}\subset H_{1}$, $i=1, \ldots, M$, $D_{j}\subset H_{2}$, $j=1, \ldots, N$, *be nonempty*, *closed*, *and convex*, *and* $L:H_{1}\rightarrow H_{2}$ *be a bounded linear operator such that* $( \bigcap_{i=1}^{M}C_{i} ) \cap L^{-1} ( \bigcap_{j=1}^{N}D_{j} ) \neq \emptyset $. *Let* $x_{1}\in H_{1}$ *and the sequence* $\{x_{n}\}$ *be generated by the following algorithm*:

$$\begin{aligned} \textstyle\begin{cases} y_{j,n}=x_{n}+\lambda_{n} L^{*}(P_{D_{j}}-I)Lx_{n}, \quad j=1, \ldots, N, \\ \textit{choose} \quad j_{n}: \Vert y_{j_{n},n}-x_{n} \Vert =\max_{j=1, \ldots, N} \Vert y_{j,n}-x _{n} \Vert , \\ y_{n}=y_{j_{n},n}, \\ z_{i,n}=P_{C_{i}}y_{n}, \quad i=1, \ldots, M, \\ \textit{choose} \quad i_{n}: \Vert z_{i_{n},n}-x_{n} \Vert =\max_{i=1, \ldots, M} \Vert z_{i,n}-x _{n} \Vert , \\ x_{n+1}=z_{i_{n},n}. \end{cases}\displaystyle \end{aligned}$$

*If* $0< a\leq \lambda_{n}\leq b<\frac{1}{2\|L\|^{2}}$ *for each* $n\in \mathbb{N}$, *then* $\{x_{n}\}$ *converges weakly to a point* $p\in ( \bigcap_{i=1}^{M}C_{i} ) \cap L^{-1} ( \bigcap_{j=1}^{N}D _{j} ) $.

#### Corollary 5.5

*Let* $H_{1}$ *and* $H_{2}$ *be real Hilbert spaces*, $C_{i}\subset H_{1}$, $i=1, \ldots, M$, $D_{j}\subset H_{2}$, $j=1, \ldots, N$, *be nonempty*, *closed*, *and convex*, *and* $L:H_{1}\rightarrow H_{2}$ *be a bounded linear operator such that* $\mathcal{F}= ( \bigcap_{i=1}^{M}C_{i} ) \cap L^{-1} ( \bigcap_{j=1}^{N}D_{j} ) \neq \emptyset $. *Let* $x_{1}\in H_{1}$ *and the sequence* $\{x_{n}\}$ *be generated by the following algorithm*:

$$\begin{aligned} \textstyle\begin{cases} y_{j,n}=x_{n}+\lambda_{n} L^{*}(P_{D_{j}}-I)Lx_{n}, \quad j=1, \ldots, N, \\ \textit{choose} \quad j_{n}: \Vert y_{j_{n},n}-x_{n} \Vert =\max_{j=1, \ldots, N} \Vert y_{j,n}-x _{n} \Vert , \\ y_{n}=y_{j_{n},n}, \\ z_{i,n}=P_{C_{i}}y_{n}, \quad i=1, \ldots, M, \\ \textit{choose} \quad i_{n}: \Vert z_{i_{n},n}-x_{n} \Vert =\max_{i=1, \ldots, M} \Vert z_{i,n}-x _{n} \Vert , \\ z_{n}=z_{i_{n},n}, \\ C_{n+1}= \{ z\in C_{n}: \Vert z_{n}-z \Vert \leq \Vert y_{n}-z \Vert \leq \Vert x_{n}-z \Vert \}, \\ x_{n+1}=P_{C_{n+1}}x_{1}. \end{cases}\displaystyle \end{aligned}$$

*If* $0< a\leq \lambda_{n}\leq b<\frac{1}{2\|L\|^{2}}$ *for each* $n\in \mathbb{N}$, *then* $\{x_{n}\}$ *converges strongly to* $q=P_{ \mathcal{F}}(x_{1})$.

### Multiple sets split equilibrium problems

Now, we apply Theorem 3.5 for getting a common solution of multiple sets split equilibrium problems. In this respect, let *C* be a nonempty closed convex subset of a Hilbert space $H_{1}$ and $F\colon C\times C\rightarrow \mathbb{R}$ be a bifunction. The equilibrium problem for bifunction *F* is the problem of finding a point $z\in H_{1}$ such that

5.2 $$\begin{aligned} F(z,y)\geq 0, \quad \forall y\in C. \end{aligned}$$

The set of solutions of equilibrium problem (5.2) is denoted by $EP(F)$. The bifunction $F\colon C\times C\rightarrow \mathbb{R}$ is called monotone if $F(x,y)+F(y,x)\leq 0$ for all $x,y\in C$. For finding a solution of equilibrium problem (5.2), we assume that *F* satisfies the following properties:

1. $F(x, x)=0$ for all $x\in C$;
2. *F* is monotone;
3. for each $x, y, z \in C$, $\limsup_{t\downarrow 0} F(tz+(1-t)x, y) \leq F(x, y)$;
4. for each $x\in C$, $y\mapsto F(x,y)$ is convex and lower semi-continuous.

Then we have the following lemma which can be found in [40, 41].

#### Lemma 5.6

*Let* *C* *be a nonempty closed convex subset of a Hilbert space* $H_{1}$ *and* $F\colon C\times C\rightarrow \mathbb{R}$ *be a bifunction satisfying properties* (*A*1)*–*(*A*4). *Let r be a positive real number and* $x\in H_{1}$. *Then there exists* $z\in C$ *such that*

$$\begin{aligned} F(z, y) +\frac{1}{r} \langle y-z, z-x\rangle \geq 0, \quad \forall y\in C. \end{aligned}$$

*Further*, *define*

$$\begin{aligned} T_{r}x= \biggl\lbrace z\in C: F(z, y) +\frac{1}{r} \langle y-z, z-x \rangle \geq 0, \forall y\in C \biggr\rbrace \end{aligned}$$

*for all* $r >0$ *and* $x\in H_{1}$. *Then the following hold*:

- $T_{r}$ *is single*-*valued*;
- $T_{r}$ *is firmly nonexpansive*; *that is*,
  $$\begin{aligned} \Vert T_{r}x-T_{r}y \Vert ^{2}\leq \langle T_{r}x-T_{r}y, x-y\rangle, \quad \forall x,y\in H_{1}; \end{aligned}$$
- $F(T_{r}) = EP (F)$;
- $EP(F)$ *is closed and convex*.

Let $C_{i}$, $i=1, \ldots, M$, and $D_{j}$, $j=1, \ldots, N$, be nonempty, closed, and convex subsets of real Hilbert spaces $H_{1}$ and $H_{2}$, respectively, $f_{i}\colon C_{i}\times C_{i} \rightarrow \mathbb{R}$, $i=1, \ldots, M$, and $g_{j}\colon D_{j} \times D_{j}\rightarrow \mathbb{R}$, $j=1, \ldots, N$, be bifunctions which satisfy properties (A1)–(A4), and $L:H_{1}\rightarrow H_{2}$ be a bounded linear operator. From Lemma 5.6 there exist the sequences $\{z_{i,n}\}$ of $H_{1}$ and $\{u_{j,n}\}$ of $H_{2}$ satisfying

5.3 $$\begin{aligned} \textstyle\begin{cases} r F_{j}(u_{j,n},y)+\langle y-u_{j,n}, u_{j,n}-Lx_{n}\rangle \geq 0, \quad \forall y\in D_{j}, j=1, \ldots, N, \\ y_{j,n}=x_{n}+\lambda_{n} L^{*}(u_{j,n}-Lx_{n}), \quad j=1, \ldots, N, \\ \mbox{choose} \quad j_{n}: \Vert y_{j_{n},n}-x_{n} \Vert =\max_{j=1, \ldots, N} \Vert y_{j,n}-x _{n} \Vert , \\ y_{n}=y_{j_{n},n}, \\ \beta_{n} F_{i}(z_{i,n},u)+\langle u-z_{i,n}, z_{i,n}-y_{n}\rangle \geq 0, \quad \forall u\in C_{i}, i=1, \ldots, M, \\ \mbox{choose} \quad i_{n}: \Vert z_{i_{n},n}-x_{n} \Vert =\max_{i=1, \ldots, M} \Vert z_{i,n}-x _{n} \Vert , \\ x_{n+1}=z_{i_{n},n}. \end{cases}\displaystyle \end{aligned}$$

Therefore, by applying Theorem 3.5, we have the following theorem for multiple sets split equilibrium problem.

#### Theorem 5.7

*Let* $C_{i}$, $i=1, \ldots, M$, *and* $D_{j}$, $j=1, \ldots, N$, *be nonempty*, *closed*, *and convex subsets of real Hilbert spaces* $H_{1}$ *and* $H_{2}$, *respectively*, $f_{i}\colon C_{i}\times C_{i} \rightarrow \mathbb{R}$, $i=1, \ldots, M$, *and* $g_{j}\colon D_{j} \times D_{j}\rightarrow \mathbb{R}$, $j=1, \ldots, N$, *be bifunctions which satisfy properties* (*A*1)*–*(*A*4). *Suppose that* $L:H_{1}\rightarrow H_{2}$ *is a bounded linear operator such that* $\mathcal{F}= ( \bigcap_{i=1} ^{M}EP(f_{i}) ) \cap L^{-1} ( \bigcap_{j=1}^{N}EP(F_{j}) ) \neq \emptyset $. *If* $\beta_{n}>0$, $0< a\leq \lambda_{n}\leq b<\frac{1}{2 \|L\|^{2}}$ *for each* $n\in \mathbb{N}$ *and* *r* *is a positive real number*, *then the sequence* $\{x_{n}\}$ *generated by* (5.3) *converges weakly to a solution of multiple sets split equilibrium problem*.

We also have the following strong convergence theorem for finding a solution of multiple sets split equilibrium problem.

#### Theorem 5.8

*Let* $C_{i}$, $i=1, \ldots, M$, *and* $D_{j}$, $j=1, \ldots, N$, *be nonempty*, *closed*, *and convex subsets of real Hilbert spaces* $H_{1}$ *and* $H_{2}$, *respectively*, $f_{i}\colon C_{i}\times C_{i} \rightarrow \mathbb{R}$, $i=1, \ldots, M$, *and* $g_{j}\colon D_{j} \times D_{j}\rightarrow \mathbb{R}$, $j=1, \ldots, N$, *be bifunctions which satisfy properties* (*A*1)*–*(*A*4). *Suppose that* $L:H_{1}\rightarrow H_{2}$ *is a bounded linear operator such that* $\mathcal{F}= ( \bigcap_{i=1} ^{M}EP(f_{i}) ) \cap L^{-1} ( \bigcap_{j=1}^{N}EP(F_{j}) ) \neq \emptyset $. *Suppose that* $x_{1}\in C_{1}=H_{1}$ *and the sequence* $\{x_{n}\}$ *is generated by the following algorithm*:

5.4 $$\begin{aligned} \textstyle\begin{cases} r F_{j}(u_{j,n},y)+\langle y-u_{j,n}, u_{j,n}-Lx_{n}\rangle \geq 0, \quad \forall y\in D_{j}, j=1, \ldots, N, \\ y_{j,n}=x_{n}+\lambda_{n} L^{*}(u_{j,n}-Lx_{n}),\quad j=1, \ldots, N, \\ \textit{choose}\quad j_{n}: \Vert y_{j_{n},n}-x_{n} \Vert =\max_{j=1, \ldots, N} \Vert y_{j,n}-x _{n} \Vert , \\ y_{n}=y_{j_{n},n}, \\ \beta_{n} F_{i}(z_{i,n},u)+\langle u-z_{i,n}, z_{i,n}-y_{n}\rangle \geq 0, \quad \forall u\in C_{i}, i=1, \ldots, M, \\ \textit{choose}\quad i_{n}: \Vert z_{i_{n},n}-x_{n} \Vert =\max_{i=1, \ldots, M} \Vert z_{i,n}-x _{n} \Vert , \\ z_{n}=z_{i_{n},n}, \\ C_{n+1}= \{ z\in C_{n}: \Vert z_{n}-z \Vert \leq \Vert y_{n}-z \Vert \leq \Vert x_{n}-z \Vert \}, \\ x_{n+1}=P_{C_{n+1}}x_{1}. \end{cases}\displaystyle \end{aligned}$$

*If* $\beta_{n}>0$, $0< a\leq \lambda_{n}\leq b<\frac{1}{2\|L\|^{2}}$ *for each* $n\in \mathbb{N}$ *and* *r* *is a positive real number*, *then the sequence* $\{x_{n}\}$ *converges strongly to* $q=P_{\mathcal{F}}(x_{1})$.

## Numerical experiments

In this section, we show some numerical examples and discuss the possible good choices of step size parameters $\beta_{n}$ and $\lambda_{n}$, which satisfy the control conditions in Theorem 3.5.

Let $H_{1}=\mathbb{R}^{2}$ and $H_{2}=\mathbb{R}^{3}$ be equipped with the Euclidean norm. Let $a_{1}:= \Bigl ({\scriptsize\begin{matrix}{} -\frac{2}{\sqrt{5}} \cr -\frac{1}{\sqrt{5}} \end{matrix}} \Bigr ) $, $a_{2}:= \Bigl ({\scriptsize\begin{matrix}{} -\frac{1}{\sqrt{2}} \cr -\frac{1}{\sqrt{2}} \end{matrix}} \Bigr ) $, and $u:= \Bigl ({\scriptsize\begin{matrix}{} -1 \cr -1 \end{matrix}} \Bigr ) $ be fixed in $H_{1}$, and $\gamma_{1}:=\cos \frac{7\pi }{18}$ and $\gamma_{2}:=\cos \frac{\pi }{3}$ be scalars. Set $\tilde{C}_{1}:=C _{1}+u$ and $\tilde{C}_{2}:=C_{2}+u$, where $C_{1}$ and $C_{2}$ are the following closed convex ice-cream cones in $H_{1}$:

$$\begin{aligned}& C_{1} := \bigl\{ x\in H_{1} : \langle a_{1}, x \rangle \geq \gamma_{1} \Vert x \Vert \bigr\} , \\& C_{2} := \bigl\{ x\in H_{1} : \langle a_{2}, x \rangle \geq \gamma_{2} \Vert x \Vert \bigr\} . \end{aligned}$$

We will consider 1-ism operators $P_{\tilde{C}_{1}}$ and $P_{ \tilde{C}_{2}}$, where $\tilde{C}_{1}$ and $\tilde{C}_{2}$ are defined by the above settings.

Next, for each $x:= \Bigl ({\scriptsize\begin{matrix}{} x_{1} \cr x_{2} \end{matrix}} \Bigr ) \in H_{1} $, we are also concerned with the following two norms:

$$ \Vert x \Vert _{1}= \vert x_{1} \vert + \vert x_{2} \vert \quad \text{and} \quad \Vert x \Vert _{\infty }= \max \bigl\{ \vert x_{1} \vert , \vert x_{2} \vert \bigr\} . $$

Consider a function $f:H_{1}\rightarrow \mathbb{R}$, which is defined by

$$ f(x)= \Vert x \Vert _{1} \quad \text{for all } x\in H_{1}. $$

We know that *f* is a convex function and subdifferential of *f* is

$$\begin{aligned} \partial f(x)= \bigl\{ z\in H_{1}:\langle x,z\rangle = \Vert x \Vert _{1}, \Vert z \Vert _{\infty }\leq 1 \bigr\} \quad \text{for all } x\in H_{1}. \end{aligned}$$

Moreover, since *f* is a convex function, we know that $\partial f( \cdot)$ must be a maximal monotone operator, and for each $\lambda >0$, we have

$$ J_{\lambda }^{\partial f}(x)= \left \{ \left ( \begin{matrix} u_{1} \\ u_{2} \end{matrix} \right ) \in H_{1}: u_{i}= x_{i}- \bigl(\min \bigl\{ \vert x_{i} \vert , \lambda \bigr\} \bigr)\operatorname{sgn}(x_{i}), \text{for } i=1, 2 \right \} , $$

where $\operatorname{sgn}(\cdot)$ is denoted for the signum function.

On the other hand, let $\tilde{x}_{1}:= \Bigl ({\scriptsize\begin{matrix}{} 1 \cr 2 \cr -1 \end{matrix}} \Bigr ) $, $\tilde{x}_{2}:= \Bigl ({\scriptsize\begin{matrix}{} -1 \cr 1 \cr -1 \end{matrix}} \Bigr ) $, and $\tilde{x}_{3}:= \Bigl ({\scriptsize\begin{matrix}{} 0 \cr -1 \cr 0 \end{matrix}} \Bigr ) $ be three fixed vectors in $H_{2}$. We consider a nonempty convex subset $Q_{1}\cap Q_{2}\cap Q_{3}$ of $H_{2}$, where $Q_{1}:=\{x \in H_{2}:\Vert \tilde{x}_{1}-x\Vert \leq 5\}$, $Q_{2}:=\{x\in H_{2}: \langle \tilde{x}_{2},x\rangle \leq 1\}$, and $Q_{3}:= \{x\in H _{2}:\langle \tilde{x}_{3},x\rangle \leq -\frac{1}{2} \}$. We notice that $F(P_{Q_{1}})\cap F(P_{Q_{2}})\cap F(P_{Q_{3}})= Q_{1}\cap Q_{2} \cap Q_{3}$.

Now, let us consider a $3\times 2$ matrix $L:= \Bigl [{\scriptsize\begin{matrix}{} 1 & 0 \cr 2 & -2 \cr 0 & 2 \end{matrix}} \Bigr ] $. We see that *L* is a bounded linear operator on $H_{1}$ into $H_{2}$ with $\Vert L\Vert =3.282073$.

Based on the above settings, we will present some numerical experiments to show the efficiency of the constructed algorithm (3.1). That is, we are going to show that algorithm (3.1) converges to a point $p\in H_{1}$ such that

6.1 $$\begin{aligned} p\in \bigl((P_{\tilde{C}_{1}}+\partial f)^{-1}(0)\cap (P_{\tilde{C} _{2}}+\partial f)^{-1}(0) \bigr)\cap L^{-1} (Q_{1}\cap Q_{2}\cap Q_{3}), \end{aligned}$$

and in this experiment, we consider the stopping criterion by $\frac{\|x_{n+1}-x_{n}\|}{\max \{1,\|x_{n}\|\}}\leq 1.0e^{-06}$.

We will consider the following cases of the step size parameters $\beta_{n}$ and $\lambda_{n}$ with the initial vectors $\Bigl ({\scriptsize\begin{matrix}{} 0 \cr 0 \end{matrix}} \Bigr ) $, $\Bigl ({\scriptsize\begin{matrix}{} 1 \cr 1 \end{matrix}} \Bigr ) $, $\Bigl ({\scriptsize\begin{matrix}{} 1 \cr -1 \end{matrix}} \Bigr ) $, $\Bigl ({\scriptsize\begin{matrix}{} -1 \cr 1 \end{matrix}} \Bigr ) $, and $\Bigl ({\scriptsize\begin{matrix}{} -1 \cr -1 \end{matrix}} \Bigr ) $ in $H_{1}$:

Case 1.: $\beta_{n}=1.0e^{-03}+\frac{1}{100n}$, $\lambda_{n}=1.0e ^{-03}+\frac{1}{100n}$.
Case 2.: $\beta_{n}=1.0e^{-03}+\frac{1}{100n}$, $\lambda_{n}=\frac{1}{4 \|L\|^{2}}$.
Case 3.: $\beta_{n}=1.0e^{-03}+\frac{1}{100n}$, $\lambda_{n}=0.046- \frac{1}{100n}$.
Case 4.: $\beta_{n}=1$, $\lambda_{n}=1.0e^{-03}+\frac{1}{100n}$.
Case 5.: $\beta_{n}=1$, $\lambda_{n}=\frac{1}{4\|L\|^{2}}$.
Case 6.: $\beta_{n}=1$, $\lambda_{n}=0.046-\frac{1}{100n}$.
Case 7.: $\beta_{n}=1.999-\frac{1}{100n}$, $\lambda_{n}=1.0e^{-03}+ \frac{1}{100n}$.
Case 8.: $\beta_{n}=1.999-\frac{1}{100n}$, $\lambda_{n}=\frac{1}{4 \|L\|^{2}}$.
Case 9.: $\beta_{n}=1.999-\frac{1}{100n}$, $\lambda_{n}=0.046- \frac{1}{100n}$.

From Tables 1, 2, and 3, we may suggest that, for each initial point, the step size of the parameters $\lambda_{n}=0.046-\frac{1}{100n}$ provides a faster convergence rate than other cases. While the step size parameters $\beta_{n}$ seem to have less impact on the speed of algorithm (3.1) to a solution set (6.1).

**Table 1:** Influence of the step size parameters $\beta_{n}$ and $\lambda_{n}$ (cases 1–3) of algorithm (3.1) for different initial points

Case →	Case 1			Case 2			Case 3		
#Initial point ↓	Iters	Time (s)	Sol	Iters	Time (s)	Sol	Iters	Time (s)	Sol
$(0,0)^{\top }$	1647	0.644764	$\Bigl ({\scriptsize\begin{matrix}{} 0.249753\cr0\end{matrix}} \Bigr )$	145	0.210611	$\Bigl ({\scriptsize\begin{matrix}{} 0.249990\cr0\end{matrix}} \Bigr )$	110	0.172755	$\Bigl ({\scriptsize\begin{matrix}{} 0.249996\cr0\end{matrix}} \Bigr )$
$(1,1)^{\top }$	790	0.393530	$\Bigl ({\scriptsize\begin{matrix}{} 1.124877\cr0.875123\end{matrix}} \Bigr )$	51	0.117471	$\Bigl ({\scriptsize\begin{matrix}{} 1.124996\cr0.875004\end{matrix}} \Bigr )$	27	0.098625	$\Bigl ({\scriptsize\begin{matrix}{} 1.124997\cr0.875001\end{matrix}} \Bigr )$
$(1,-1)^{\top }$	195	0.231496	$\Bigl ({\scriptsize\begin{matrix}{} 0.875676\cr0\end{matrix}} \Bigr )$	49	0.123486	$\Bigl ({\scriptsize\begin{matrix}{} 0.795371\cr0\end{matrix}} \Bigr )$	36	0.127907	$\Bigl ({\scriptsize\begin{matrix}{} 0.787096\cr0\end{matrix}} \Bigr )$
$(-1,1)^{\top }$	1069	0.486436	$\Bigl ({\scriptsize\begin{matrix}{} 0.267956\cr0.018131\end{matrix}} \Bigr )$	150	0.207209	$\Bigl ({\scriptsize\begin{matrix}{} 0.249990\cr0\end{matrix}} \Bigr )$	113	0.181702	$\Bigl ({\scriptsize\begin{matrix}{} 0.249996\cr0\end{matrix}} \Bigr )$
$(-1,-1)^{\top }$	2121	0.847208	$\Bigl ({\scriptsize\begin{matrix}{} 0.249752\cr0\end{matrix}} \Bigr )$	449	0.313106	$\Bigl ({\scriptsize\begin{matrix}{} 0.249991\cr0\end{matrix}} \Bigr )$	361	0.284821	$\Bigl ({\scriptsize\begin{matrix}{} 0.249996\cr0\end{matrix}} \Bigr )$

**Table 2:** Influence of the step size parameters $\beta_{n}$ and $\lambda_{n}$ (cases 4–6) of algorithm (3.1) for different initial points

Case →	Case 4			Case 5			Case 6		
#Initial point ↓	Iters	Time (s)	Sol	Iters	Time (s)	Sol	Iters	Time (s)	Sol
$(0,0)^{\top }$	1647	0.650587	$\Bigl ({\scriptsize\begin{matrix}{} 0.249753\cr0\end{matrix}} \Bigr )$	106	0.176374	$\Bigl ({\scriptsize\begin{matrix}{} 0.249991\cr0\end{matrix}} \Bigr )$	56	0.124235	$\Bigl ({\scriptsize\begin{matrix}{} 0.249996\cr0\end{matrix}} \Bigr )$
$(1,1)^{\top }$	790	0.398679	$\Bigl ({\scriptsize\begin{matrix}{} 1.124877\cr0.875123\end{matrix}} \Bigr )$	51	0.122999	$\Bigl ({\scriptsize\begin{matrix}{} 1.124996\cr0.875004\end{matrix}} \Bigr )$	27	0.098005	$\Bigl ({\scriptsize\begin{matrix}{} 1.124999\cr0.875001\end{matrix}} \Bigr )$
$(1,-1)^{\top }$	3	0.078350	$\Bigl ({\scriptsize\begin{matrix}{} 0.985333\cr0\end{matrix}} \Bigr )$	3	0.079696	$\Bigl ({\scriptsize\begin{matrix}{} 0.969096\cr0\end{matrix}} \Bigr )$	3	0.083422	$\Bigl ({\scriptsize\begin{matrix}{} 0.952000\cr0\end{matrix}} \Bigr )$
$(-1,1)^{\top }$	1032	0.500529	$\Bigl ({\scriptsize\begin{matrix}{} 0.575413\cr0.325587\end{matrix}} \Bigr )$	61	0.133658	$\Bigl ({\scriptsize\begin{matrix}{} 0.520560\cr0.270565\end{matrix}} \Bigr )$	31	0.108214	$\Bigl ({\scriptsize\begin{matrix}{} 0.462999\cr0.213001\end{matrix}} \Bigr )$
$(-1,-1)^{\top }$	1658	0.689241	$\Bigl ({\scriptsize\begin{matrix}{} 0.249753\cr0\end{matrix}} \Bigr )$	107	0.180100	$\Bigl ({\scriptsize\begin{matrix}{} 0.249991\cr0\end{matrix}} \Bigr )$	57	0.129912	$\Bigl ({\scriptsize\begin{matrix}{} 0.249996\cr0\end{matrix}} \Bigr )$

**Table 3:** Influence of the step size parameters $\beta_{n}$ and $\lambda_{n}$ (cases 7–9) of algorithm (3.1) for different initial points

Case →	Case 7			Case 8			Case 9		
#Initial point ↓	Iters	Time (s)	Sol	Iters	Time (s)	Sol	Iters	Time (s)	Sol
$(0,0)^{\top }$	1647	0.644395	$\Bigl ({\scriptsize\begin{matrix}{} 0.249753\cr0\end{matrix}} \Bigr )$	106	0.167910	$\Bigl ({\scriptsize\begin{matrix}{} 0.249991\cr0\end{matrix}} \Bigr )$	56	0.122966	$\Bigl ({\scriptsize\begin{matrix}{} 0.249996\cr0\end{matrix}} \Bigr )$
$(1,1)^{\top }$	790	0.403824	$\Bigl ({\scriptsize\begin{matrix}{} 1.124877\cr0.875123\end{matrix}} \Bigr )$	51	0.118171	$\Bigl ({\scriptsize\begin{matrix}{} 1.124996\cr0.875004\end{matrix}} \Bigr )$	27	0.095997	$\Bigl ({\scriptsize\begin{matrix}{} 1.124999\cr0.875001\end{matrix}} \Bigr )$
$(1,-1)^{\top }$	3	0.080739	$\Bigl ({\scriptsize\begin{matrix}{} 0.985333\cr0\end{matrix}} \Bigr )$	3	0.080157	$\Bigl ({\scriptsize\begin{matrix}{} 0.969096\cr0\end{matrix}} \Bigr )$	3	0.080880	$\Bigl ({\scriptsize\begin{matrix}{} 0.952000\cr0\end{matrix}} \Bigr )$
$(-1,1)^{\top }$	1032	0.463895	$\Bigl ({\scriptsize\begin{matrix}{} 0.575413\cr0.325587\end{matrix}} \Bigr )$	61	0.133494	$\Bigl ({\scriptsize\begin{matrix}{} 0.520560\cr0.270565\end{matrix}} \Bigr )$	31	0.104363	$\Bigl ({\scriptsize\begin{matrix}{} 0.462999\cr0.213001\end{matrix}} \Bigr )$
$(-1,-1)^{\top }$	1658	0.646397	$\Bigl ({\scriptsize\begin{matrix}{} 0.249753\cr0\end{matrix}} \Bigr )$	107	0.173753	$\Bigl ({\scriptsize\begin{matrix}{} 0.249991\cr0\end{matrix}} \Bigr )$	57	0.127317	$\Bigl ({\scriptsize\begin{matrix}{} 0.249996\cr0\end{matrix}} \Bigr )$

## Conclusions

In this paper, we present two iterative algorithms, (3.1) and (4.1), for approximating a solution of the split feasibility problem on zeros of a finite sum of monotone operators and fixed points of a finite family of nonexpansive mappings. Under some mild conditions, we show the convergence theorems of the mentioned algorithms. Subsequently, some corollaries and applications of those main results are provided. We point out that the construction of algorithm (3.1) seems to be less complicated than that of (4.1). However, algorithm (3.1) requires some additional assumptions in order to guarantee the strong convergence theorem, while algorithm (4.1) does not need them (see Theorem 3.6 and Theorem 4.2). This observation may lead to the future works that are to analyze and discuss the rate of convergence of these suggested algorithms.
